# Ascending aorta perforation with cardiac tamponade 19 days after transcatheter aortic valve implantation

**DOI:** 10.1007/s12471-016-0867-x

**Published:** 2016-08-29

**Authors:** R. Joustra, P. Kievit, M. Verkroost, H. Gehlmann, M.-J. de Boer

**Affiliations:** 1 Department of Cardiology, Radboud University Medical Center, Nijmegen, The Netherlands; 2 Cardiothoracic Surgery, Radboud University Medical Center, Nijmegen, The Netherlands

A 83-year-old Caucasian male, known with chronic pulmonary disease, mild renal insufficiency, hypertension and epilepsy, was admitted to our tertiary referral centre for aortic valve replacement because of symptomatic severe aortic stenosis. Echocardiography showed aortic stenosis with a peak gradient of 100 mmHg (aortic valve area 0.5 cm^2^) and a hypertrophic left ventricle with moderate systolic function (LVEF 50 %). The diameter of the ascending aorta was 33 mm on echocardiography and the aortic valve was functionally bicuspid, with tricuspid architecture and fusion of two leaflets. Coronary angiography showed coronary atherosclerosis without significant stenosis. The logistic EuroSCORE was 11.02 %. The multidisciplinary heart team considered the patient at high risk for conventional surgery because of comorbidities, frailty and age. Transcatheter aortic valve implantation (TAVI) was judged to be the optimal therapeutic option.

The TAVI procedure was performed under general anaesthesia with vascular access through the left subclavian artery, which is the access site of choice in our centre. The native valve with an annular dimension of 25 mm, as assessed by transoesophageal echocardiography, was predilatated with a 22 mm Nucleus balloon (NuMED Inc.) under rapid pacing. A 29 mm Medtronic CoreValve**®** was inserted and deployed. After implantation a mild gradient and moderate paravalvular leakage were observed, due to extensive local annular calcification. After two postdilatations of the prosthesis at the aortic valve annulus, both the aortic regurgitation and transvalvular gradient disappeared. As in the majority of TAVI centres in the Netherlands [1], the antithrombotic regimen in our patient was aspirin with clopidogrel. Postoperative recovery was uncomplicated and no conduction disorders were observed. The patient was discharged after one week with an improvement in exercise tolerance to NYHA class II.

Nineteen days after TAVI, the patient was urgently readmitted to our hospital because of acute chest pain, accompanied by severe shortness of breath and hypotension. At our emergency department we saw a critically ill patient with clinical signs of cardiac tamponade. Systolic blood pressure decreased from 100 to 60 mmHg and was restored with intravenous fluid suppletion. Urgent echocardiography showed 2 cm of pericardial effusion with right ventricular diastolic collapse and marked under-filling of the left ventricle. The diagnosis was ‘acute cardiac tamponade’, probably as a late complication of TAVI. Urgent CT showed periaortic haematoma and contrast extravasation (Fig. 1a) at the level of the distal site of the prosthesis. The distal stent strut was located outside the ascending aorta towards the pulmonary artery continuation (Fig. 1b and c). CT confirmed the echocardiographic view of pericardial effusion (Fig. 1d).

**Fig. 1:**
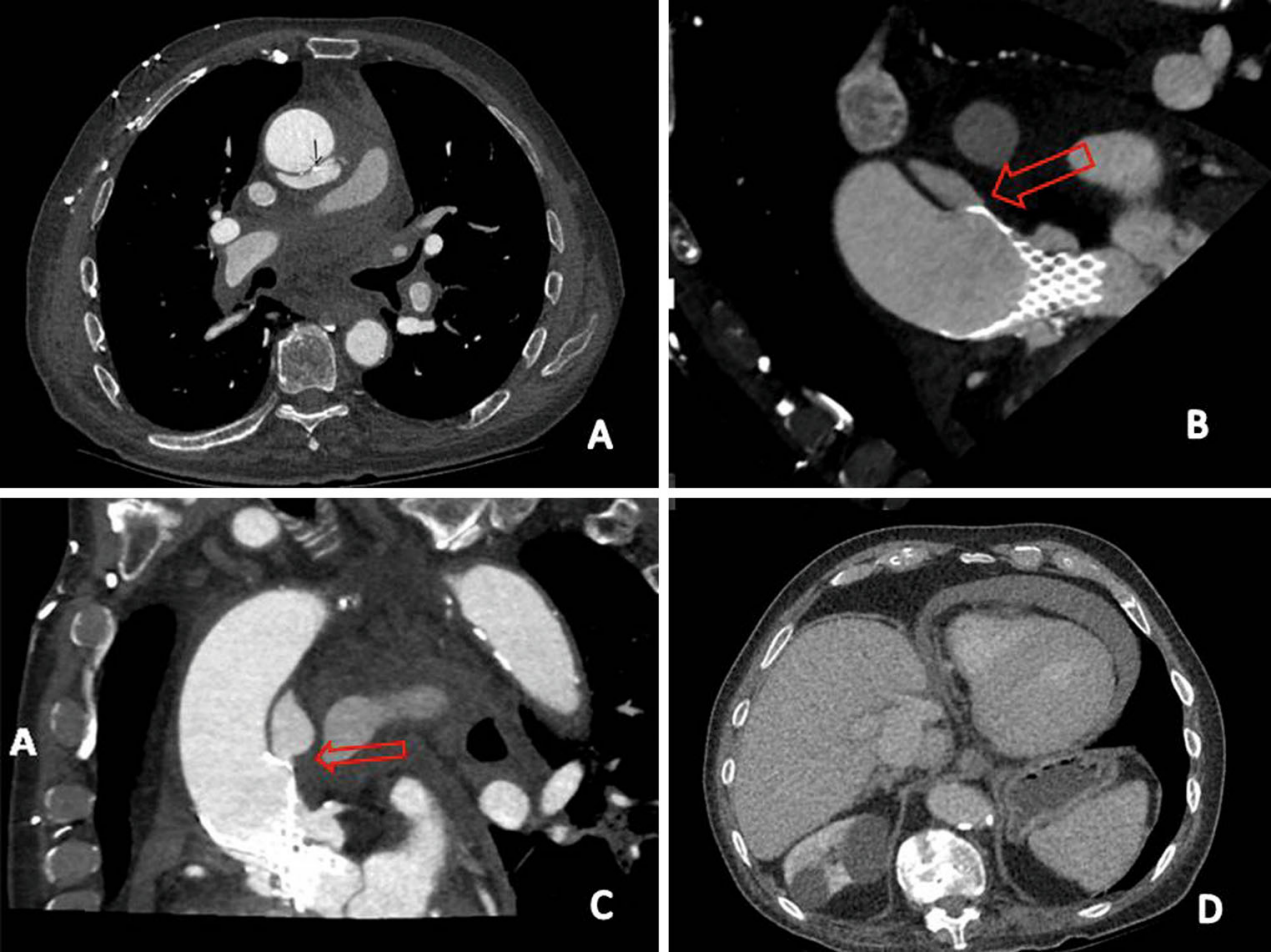
**a** periaortic haematoma and contrast extravasation at the level of the distal site of the prosthesis. **b** and **c** The distal stent strut was located outside the ascending aorta towards the pulmonary artery continuation. **d** CT confirmed the echocardiographic view of pericardial effusion

After an urgent heart team meeting and consultation with the patient and family, immediate cardiac surgery was performed. After opening the pericardium a huge aortic haematoma was found. Femoral arterial cannulation was installed. After clamping the distal ascending aorta at the level of the brachiocephalic artery a full transection of the ascending aorta was performed. A 3 cm large laceration at the inner curve was found with complete destruction of the adventitial layer. The aortic annulus and sinus had a normal aspect. After removal of the Medtronic CoreValve, a 23 mm biological aortic valve prosthesis was implanted followed by a supracoronary replacement of the ascending aorta using a 30 mm Gelweave vascular prosthesis (Terumo Vascutek). Postoperative complications were acute tubular necrosis and delirium, but both recovered well. The patient could be discharged home ten days after surgery and was already driving his car one and a half month later. This is the first reported case describing this complication with this timing.

## References

[1] Nijenhuis VJ, Stella PR, Baan J (2014). Antithrombotic therapy in patients undergoing TAVI: an overview of Dutch hospitals. Neth Heart J.

